# Prevalence of using non prescribed medications in economically deprived rural population of Pakistan

**DOI:** 10.1186/s13690-015-0113-9

**Published:** 2016-01-22

**Authors:** Abdul Haseeb, Muhammad Bilal

**Affiliations:** Dow University of Health Sciences, Karachi, Pakistan

**Keywords:** Self medication, Economically deprived, Rural population, Prevalence, Outpatient department

## Abstract

**Background:**

Self medication is described as an act of procurement and consumption of medical drugs without the advice of medical physician for diagnosis, prescription and surveillance of treatment. There is a paucity of literature with regards to self medication among rural dwellers of Pakistan and no initiatives have been taken to resolve this issue. Therefore, the study aimed to evaluate frequency, practice and prevalence of self medication among economically deprived rural population of Karachi (South Pakistan).

**Methods:**

This was the descriptive, epidemiological cross sectional survey which was conducted at the two largest tertiary care government based teaching hospitals of Karachi, Civil hospital and Jinnah Postgraduate Medical Centre, from January 2015 until March 2015. Seven hundred rural dwellers were recruited; who were the residents of outskirts of Karachi city were enrolled in the above mentioned period through the outpatient department (OPD) of the respective hospitals.

**Results:**

According to the survey, 595 (85 %) subjects practiced self medication. The most common reasons evaluated for self medication were cost of consultation (90.3 %) and availability of transport (81.0 %) from rural area to health care facility. The paracetamol as a painkiller (93.0 %), acetylsalicylic acid as an anti pyretic (69.0 %), anti biotic (52.0 %) and anti allergic (51.0 %) were the commonest drug used without prescription of a health care physician. A significant difference was obtained in carrying out self medication between participants earning less than 50,000 PKR and greater than this amount (*p* = 0.029; 61 % vs. 24 %) and for the self medicated patients having education less than graduation with the participants having education of graduation or above it (*p* = 0.03; 63 % vs. 22 %).

**Conclusion:**

The self medication among rural dwellers of Karachi is high . As a result, urgent steps must be taken to initiate the awareness and educational programs regarding potential risks of self medication. Secondly, strict measures must be introduced to stop supply of prescription drugs from pharmacies without prescription. Thirdly, provision of cost effective treatment from public sector hospitals to rural population can help to reduce self medication among rural population of Pakistan.

## Background

Self medication is described as an act of procurement and consumption of medical drugs without the advice of medical physician for diagnosis, prescription and surveillance of treatment [1]. The definition is further applicable with regards to treatment of family members, specifically children and elderly. The practice is considered as a major element of self care and primary public health resource in health care system of Pakistan. The epidemiological data has revealed high prevalence all over the world; up to 59 % in Nepal [2] and other developing countries [2], 92 % in adolescents of Kuwait [3], 31 % in India [4] and in European countries (Sweden, Finland, Netherlands) ranged from 17–67 %, depending on the underlying recall period [5]. However, few studies that were conducted in Pakistan have revealed the prevalence of 51 % [6] among children. Moreover, studies conducted in Karachi have confirmed the frequency of self medication among university students to be as high as 80.4 % [7]. Particularly, usage frequency among non-medical students was 83.3 % while for those in medical school, it was estimated to be 77.7 % [7].

Recently, there has been a considerable increased concern among public and professional health associations regarding irrational usage of drugs [8]. Studies have deduced that behaviors towards self medication are more common among women specially those who live alone, people facing chronic health issues, psychiatric conditions and those who are of younger age and are students [9]. Furthermore, lack of access to healthcare, availability of medicines as over the counter medicines, poor regulatory practices and long delay in medical care from hospitals has contributed significantly to rising trends in modern societies [10]. It is also claimed that self medication current trends are economic driven, where the person who is not able to pay for the cost of medicines practices self medication because he finds it feasible [11].

It is believed that public awareness along with enforcing and implementing laws about prescribed medications can immensely reduce the rate of self medication [12]. In Pakistan, it is a common practice that the prescription drug is sold in pharmacy without prescription; which is strictly prohibited according to local law. Pharmacist dispensing these prescribed drugs have no information of patients allergies and as a result antibiotics and habit forming medicines are easily accessible without prescription.. Furthermore, there is a paucity of literature with regards to self medication among rural dwellers of Pakistan and no initiatives have been taken to resolve this issue. Therefore, the study aimed to evaluate frequency, practice and prevalence of self medication among economically deprived rural population of Karachi (South Pakistan).

## Methods

### Study design and setting

This was the descriptive, epidemiological cross sectional survey which was conducted at the two largest tertiary care government based teaching hospitals of Karachi, Civil hospital and Jinnah Postgraduate Medical Centre, from January 2015 until March 2015. Both the hospitals are known to provide tertiary care treatment free of cost, so they are basic health care providers for rural dwellers living in the outskirts of Karachi city who cannot afford the cost of treatment at other health care setups.

### Sample size calculation

The local literature does not provide any prevalence of self medication among rural dwellers living in outskirts of Karachi. However, previous studies conducted on medical university students affiliated with tertiary care teaching hospitals of Karachi revealed a prevalence of self medication to be 75–80 % [7].

A pilot study was conducted initially to test the questionnaire on 30 subjects who fulfilled the inclusion criteria of our study in the same setting at tertiary care teaching hospital. The data collected from these participants was analyzed and proportion of the subjects that self medicated was calculated which reported to be 80 %. Moreover, the feedback was also obtained from investigators and participants. In the light of responses obtained, ambiguities from questionnaire were removed before its implementation in the final study. The data from pilot study was not included in the final results.

Ultimately, using 80 % as a reference prevalence of usage of self medication on rural dwellers of Karachi, the minimum sample size was calculated to be 693, at a confidence interval of 99.9 %. However, 700 subjects were recruited for the study.

### Inclusion and exclusion criteria

Seven hundred participants who were residents of outskirts of Karachi city area were considered as the rural dwellers and enrolled in the above mentioned period through the outpatient department (OPD) of the respective hospitals. Participants belonging to medical, paramedical and pharmacology fields were excluded from the study survey to avoid selection bias as they are well aware with potential side effects of self medication and expected to have lower self medication rates.

To meet the inclusion criteria, self medication was defined as usage of any medicinal product (prescription drugs and over-the-counter drugs as well), in the past 6 months, with one’s own accord, which was not prescribed by a health physician. A time period of 6 months was allocated to eliminate recall bias among the participants who had used antibiotics and were likely to recall it in this adequate time period, with those participants who had not used antibiotics and would also clearly remember not having used them in the suggested time period.

### Data collection procedure

A non probability convenience sampling was employed at the OPD of two tertiary care hospitals to recruit a sample size of 700. Both verbal and written informed consents were obtained from the participants after brief introduction of the study. A pre-designed and pre tested questionnaire [13] in English language was used to collect the responses. However, participants who did not understand English were interviewed by trained medical researchers in their local language Urdu and later, responses were back translated in English before entering information into questionnaire. During the data collection procedure, participants were also assessed with the indication for the reported drug used for (e.g. whether the paracetamol was used as a pain killer or anti pyretic drug).

### Date collection tool

We used paper based questionnaire which was divided into 3 parts for data collection. Each section was preceded by the statement to elaborate the type of question that would follow, for the better understanding of participants. The first part assessed the demographical features of the subject followed by second part which dealt with the prevalence and practice of self medication. Lastly, third part focused on attitudes of rural dwellers towards self medication. The entire tool consisted of close ended questions only.

### Data entry and analysis

Data entry and analysis was performed using SPSS version 19. Moreover, the descriptive analysis was performed by calculating means and proportions for continuous and discrete data respectively. Inferential analysis was conducted by employing the Pearson chi square test of significance to identify associations amongst demographical variables like gender and educational status with prevalence of self medication. A *p* value of < 0.05 was considered as statistically significant.

### Ethical consideration

The research protocol was approved by the Ethical Review Board of Dow university of Health Sciences. Additionally, permissions were obtained prior to study from respective hospitals.

## Results

A remarkably high response rate of 100 % was obtained, due to proper explanation of research proposal and assurance of maintaining full confidentiality of the data they provided. Three fifty participants (50 %) were recruited from each hospital. The mean age of the subjects was 48.5 ± 6.3, consisting of 441 (63 %) males and 259 (37 %) females. Besides, 217 (31) participants were matriculated (grade 10),182 (26) had secondary school degree (intermediate), 77 (11 %) participants were graduated,49 (7 %) had qualification of post graduation and 175 (25 %) OPD patients had no formal education. Regarding occupation, 329 (47 %) were employed,77 (11 %) were retired, 161 (23 %) were un-employed and 133 (19 %) were students. When questioned about monthly income, 147 (21 %) earned <5000 PKR, 210 (30 %) responded in the range of 5000–10,000PKR, 182 (26 %) subjects responded 10,000–20,000 PKR, 95 (13.5 %) responded 20,000–50,000PKR, 66 (9.5 %) responded for >50,000PKR. Further,429 (61.3 %) participants were married, 122 (17.5 %) were single,56 (8.0 %) were widow and 92 (13.2 %) were divorced. For the subdivision of chronic illness, 365 (52.2 %) were not having any chronic disease where as 77 (11 %) responded diabetes and 133 (19.0) claimed cardiovascular diseases.

According to the survey, 595 (85 %) subjects practiced self medication. The most common reason evaluated for self medication was cost of consultation (90.3 %), availability of transport (81.0 %), advice from friend/family (78.0 %), convenience (62.0 %) and previous experience (67.0). The prevalent symptoms that led rural dwellers to indulge in self medication were headaches (81.0 %), flu/cough (79.0 %), fever (68.0 %) and pain elsewhere in body (63.0 %). As a result paracetamol as a painkiller (93.0 %), acetylsalicylic acid as an anti pyretic (69.0 %), anti biotic (52.0 %) and anti allergic (51.0 %) were the commonest drug used without prescription of a health care physician. Besides, 90 % (535) never read the instructions that are placed with medicine and only 9 % (54) read about side effects and contraindications. Moreover, 35 % (208) of the subjects practicing self medication adjusted dosage of medicine consulting pharmacist and 22 % (131) adjusted according to previous experiences.

Furthermore, for the most prevalent self medicated drug, paracetamol (pain killers) were used with the frequency of every few months by 216 (39 %) subjects, 172 (42 %) subjects also used acetylsalicylic acid (anti pyretic) within every few months. However, anti allergics were used by 136 (45 %) participants with with the frequency of 2–3 times in a year whereas same frequency was recorded for usage of antibiotics by 151 (49 %) participants. Alarmingly, only 196 (33 %) self medicated OPD patients were aware of the fact that their practice can lead to harmful effects and only 150 (25.2 %) subjects among them appreciated that they should consult a medical physician before starting a new medicine.

Further analysis was performed to assess the significant difference associated with demographical features with regards to prevalence of self medication. There was no significant difference between self medication practiced by male and females (*p* = 0.32; 41 % vs 44 %) and subjects having a chronic disease with participants having no disease (*p* = 0.48; 42 % vs 43 %). However, a significant difference was obtained between participants earning less than 50,000 PKR and greater than this amount (*p* = 0.029; 61 % vs 24 %) and for the self medicated patients having education less than graduation with the participants having education of graduation or above it (*p* = 0.03; 63 % vs 22 %) (Tables 1, 2, 3 and 4).

**Table 1:** Illustrates demographic features of study participants

Variable	*N* = 700	%
Age ± SD	48 ± 6.3	
Gender		
Male	441	63.0
Female	259	37.0
Education		
Matric	217	31.0
Intermediate	182	26.0
Graduation	77	11.0
Post graduation	49	7.0
No education	175	25.0
Occupation		
Working	329	47.0
Retired	77	11.0
Un-employed	161	23.0
Student	133	19.0
Monthly income		
<5000 PKR	147	21.0
5000–10,000	210	30.0
10,000–20,000	182	26.0
20,000–50,000	95	13.5
>50,000	66	9.5
Marital status		
Single	122	17.5
Married	429	61.3
Widow	56	8.0
Divorce	92	13.2
Type of chronic illness		
Thyroid Disorder	24	3.4
Diabetes	77	11.0
Hypertension	52	7.4
Hyperlipidemia	6	0.9
Cardiovascular	133	19.0
Bone illness	42	6.0
None	365	52.2

**Table 2:** Illustrates factors and symptomatology that leads to self medication among study participants

Variable	*N* = 595	%
Perceived reasons for self medication among rural population		
Cost of consultation	537	90.3
Lack of availability of transport	482	81.0
Advice from friend/family was enough	464	78.0
Previous experience	399	67.0
Convenience	369	62.0
Home stock	280	47.0
Problem too trivial	268	45.0
Urgency of problem	232	39.0
Public pharmacy	208	35.0
Lack of time	48	8.0
Symptomatology leading to self medication		
Headaches	482	81.0
Flu/cough/cold	470	79.0
Fever	405	68.0
Pain elsewhere	375	63.0
Diarrhea	244	41.0
Allergy	202	33.9
Inability to sleep	149	25.0
Blood pressure	125	21.0
Diabetes	113	19.0

**Table 3:** Illustrates attitudes of study participants towards self medication

Variable	*N* = 595	%
Commonest drugs used to self medicate		
Paracetamol (Pain killers)	553	93.0
Aspirin (Fever relieving medicine)	410	69.0
Anti allergy	303	51.0
Antibiotics	309	52.0
Vitamins	250	42.0
Pills for indigestion	148	24.5
Sleeping pills	173	29.0
Herbal/homeopathic	107	18.0
Tonics	54	9.0
Street drugs	35	5.8
Birth control pills	30	5.0
Reading Medication Instructions		
Side Effects	54	9.0
Contraindications	54	9.0
Doses	60	10.0
All	42	7.0
Indication	54	9.0
Never read medical instructions	535	90.0
Dosage of Medication		
Pharmacist	208	35.0
Previous doses	131	22.0
According to pain	65	11.0
According to drug instruction	48	8.0
Severity	65	11.0
Randomly	89	15.0

**Table 4:** Depicts frequencies of most common self medicated drugs

Frequency	Pain killers	Anti pyretics	Anti allergics	Antibiotics
Once	72 (13 %)	45 (11 %)	45 (15 %)	65 (21 %)
2–3 times/yr	111 (20 %)	90 (22 %)	136 (45 %)	151 (49 %)
Every few months	216 (39 %)	172 (42 %)	76 (25 %)	59 (19 %)
Every few weeks	105 (19 %)	70 (17 %)	30 (10 %)	22 (7 %)
All the time	50 (9 %)	33 (8 %)	15 (5 %)	12 (4 %)

## Discussion

The current study highlights that about 85 % population of rural dwellers of Karachi self medicate. To the best of our knowledge this is the first study of its kind and there is no such data available on rural dwellers of other cities, as a result there is no study available for comparison on national level. However, a study conducted on Pakistani mothers reported a prevalence of 51 % for giving medicines to their children without consulting medical doctor in a district of Karachi [6]. Coversely, a study indicated that 76 % of university students of Karachi self medicate [13]. The results of our study are in line with the situation of Croatia [14] and Hong Kong [15] where percentage population found to be self medicating were 88 % and 94 % respectively from results of 2001. However our results are inconsistent with the work of Deshpande SG et al., who has reported the prevalence of self medication of 31 % in general population of India [4]. This suggests that prevalence calculated in our study is quite high and serious actions must be taken by health care authorities to tackle the situation.

It is also worthy to consider the fact that self medication can help to cure minor sickness that do not require advice of medical doctor and it also reduces the burden on country’s health department specifically in the developing and underdeveloped countries having limited health care resources [14]. However, the ease of accessibility of more complex drugs such as antibiotics without prescription has become a source of growing concern [16]. The increase usage of non prescribed drugs has many adverse effects and can lead to global health concerns like emergence of Multi-Drug resistant pathogenisis [19], drug dependence and addiction [20], masking of malignant and lethal diseases [17, 18], danger of misdiagnosis [21], problems relating to over and under dosaging [19], drug interactions with other compounds in body [22] and problems relating to the side effect profile of particular drugs [23].

Among rural dwellers of Karachi, the most popular reasons behind self medication were cost of consulting a medical doctor (90 %), lack of facility of transport (81 %) and advice from friend/relative (78 %) . This is due to the fact that owing to low socioeconomic status and lack of infrastructure, rural dwellers are inaccessible to medical facilities available in nearby cities. However, results are contradictory with the other study conducted on university students of Pakistan [13] where the most prevalent response was ‘previous experience with similar symptoms’ (50.3 %) and the ‘problem seeming to be too trivial’ (48.3 %). Another study conducted in Saudi Arab; Majmaah city also reported previous experience with similar symptoms to be the most commonest answer for self medication [24].

Furthermore, results also revealed that medicines were directly bought from pharmacies and alarmingly, usage of stock of medicines present at home was the most popular option among rural dwellers for the availability of drugs to self medicate. The latter induces the higher risk of exposure to expired medicines, medicines brought for someone else at home or drugs that have been advised for different health issues; as a result this practice could lead to fatal side effects [14]. The former factor is valid due to the fact that all kinds of drugs including prescribed medicines are easily available from pharmacies and medical stores without prescription which is a common practice in Pakistan. Strict vigilance must be imposed on selling of medicines that are not categorized as over-the-counter drug by government bodies in order to stop this practice. On the other hand, various studies have revealed that easy access to pharmaceuticals have become an important determinant of self medication in our societies [8]. This draws our attention to increase marketing and advertisements carried out by pharmaceutical companies through print and electronic media. Researchers have indicated that advertisements lead to individuals decision to self medicate [25]. As a result strict monitoring policies must be introduced to resolve the issue. Marketing and advertisements by pharmaceutical companies should be restricted to over-the-counter drugs only.

Our results also demonstrated that the most common drugs used to self medicate were anti pyretic, anti allergic, pain killer and anti biotic to treat fever, pain, flu, headache and allergies respectively. The outcome is similar with the study conducted in Majmaah [24]. The results are alarming as 52 % of those who self medicated used anti biotic with half of the consumers used at a frequency of 2–3 times a year . The numbers are higher than the study conducted in Pakistan on university students [13]. The results are comparable to a study conducted in Southern Spain where significant number of people employed anti biotic without proper clinical evaluation [26]. The serious health issue linked with this indiscriminate use is development of anti biotic resistance in Pakistan [17]. The results derived from this study can help to provide a framework for educational programs that will create knowledge about the risk of self-prescribed antibiotics in Pakistan.

Strikingly, 90 % of those who self medicated never red medications instructions and quite a few of people adjusted dosage of medicine according to drug instructions. The results could be attributed to the low educational qualification of rural dwellers. Therefore, vigorous media campaigns must be initiated by government and private bodies to emphasize people regarding importance of reading medication instructions and adverse effects associated with ill adjustment of dosage of medicine.

There were few limitations to the study. Firstly, it was only conducted on rural dwellers of Karachi, therefore results could not be generalized to entire population of Pakistan. Secondly, it was a cross sectional survey, therefore it might have excluded causal relationships between the dependent and independent variable . Thirdly, it was a self administered questionnaire for most of the participants, therefore they might have over reported socially acceptable behaviors and under reported socially undesirable responses. There were no methods to employ in order to evaluate the honesty of participants’ answers to the question in survey. The absence of identifying data on the survey sheets and confidentiality would have helped to eliminate such bias. Moreover, it has to be considered that only those rural dwellers were recruited who visited hospital for an acute or chronic disorder, and compared with the general rural population they are likely to have used more self medication during the last 6 months. Lastly, questionnaires were filled in outpatient department and it is a possibility that subjects might have red recommended guidelines and thus acquired knowledge prior to survey. As a result we might have overestimated knowledge for few participants. Despite of the limitations discussed above, our results extended previous works and has comprehensively evaluated the self medication prevalence and attitudes among rural dwellers of Karachi.

## Conclusion

The self medication among rural dwellers of Karachi is high. As a result, urgent steps must be taken to initiate the awareness and educational programs regarding potential risk factors of self medication. Secondly, strict measures like cancellation of pharmacy permits must be implemented to stop supply of prescription drugs from pharmacies selling these medicines without prescription. Thirdly, provision of cost effective treatment from public sector hospitals to rural population can help to reduce self medication among rural population of Pakistan.
